# A sucrose-specific receptor in *Bemisia tabaci* and its putative role in phloem feeding

**DOI:** 10.1016/j.isci.2023.106752

**Published:** 2023-04-29

**Authors:** Ofer Aidlin Harari, Amir Dekel, Dor Wintraube, Yuri Vainer, Rita Mozes-Koch, Esther Yakir, Osnat Malka, Shai Morin, Jonathan D. Bohbot

**Affiliations:** 1 Department of Entomology, The Hebrew University of Jerusalem, The Robert H. Smith Faculty of Agriculture, Food and Environment, Rehovot 76100, Israel

**Keywords:** Biological sciences, Molecular biology, Molecular mechanism of behavior

## Abstract

In insects, specialized feeding on the phloem sap (containing mainly the sugar sucrose) has evolved only in some hemipteran lineages. This feeding behavior requires an ability to locate feeding sites buried deeply within the plant tissue. To determine the molecular mechanism involved, we hypothesized that the phloem-feeding whitefly *Bemisia tabaci* relies on gustatory receptor (GR)-mediated sugar sensing. We first conducted choice assays, which indicated that *B. tabaci* adults consistently choose diets containing higher sucrose concentrations. Next, we identified four *GR* genes in the *B. tabaci* genome. One of them, *BtabGR1*, displayed significant sucrose specificity when expressed in *Xenopus* oocytes. Silencing of *BtabGR1* significantly interfered with the ability of *B. tabaci* adults to discriminate between non-phloem and phloem concentrations of sucrose. These findings suggest that in phloem feeders, sugar sensing by sugar receptors might allow tracking an increasing gradient of sucrose concentrations in the leaf, leading eventually to the location of the feeding site.

## Highlights

• The phloem-feeding whitefly *Bemisia tabaci* can detect apoplastic sucrose concentrations
• One sugar receptor of *B. tabaci*, BtabGR1, displays significant sucrose specificity
• BtabGR1 is the most “narrowly tuned” sucrose receptor characterized to date
• Silencing the gene coding for BtabGR1 significantly interferes with sucrose sensing

## Graphical abstract

**Figure:**
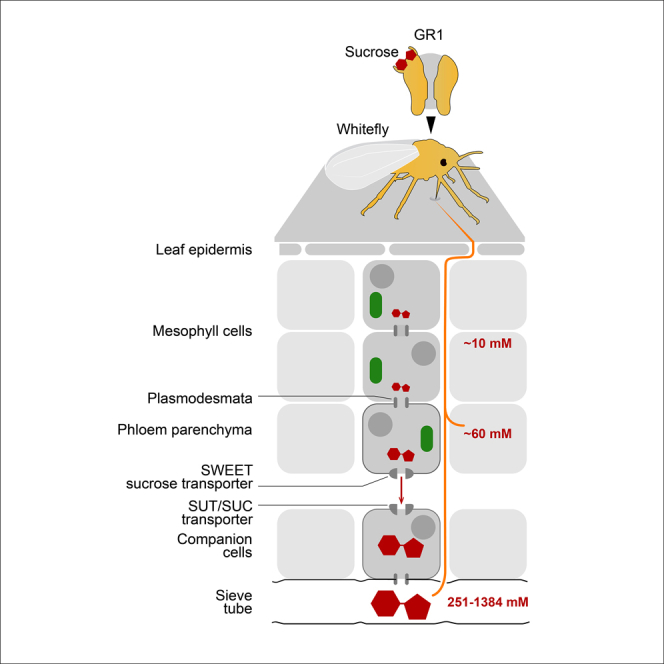

## Introduction

The phloem vascular tissue is the main pathway for long-distance transport of assimilates from source to sink organs in plants.^1^ The dominant components of the phloem sap are sugars, mainly sucrose, followed by amino acids and other nutrients.^2^ Some lineages in the order Hemiptera use the phloem sap as their dominant food source.^3^ This feeding habit is displayed by some planthoppers (suborder Auchenorrhyncha), some leafhoppers (suborder Clypeorrhyncha), and nearly all sternorrhynchan species, including whiteflies, aphids, mealybugs, and some psyllids.^3^^,^^4^^,^^5^ These insects have evolved specialized mouthparts called stylets that travel from the plant cuticle, epidermis, and mesophyll tissues to the feeding site located in the phloem sieve elements. The sensory mechanisms involved with sugar sensing in phloem-feeding insects are unknown but are likely mediated by taste organs (sensillae) on the apex of the labium, the precibarium and cibarium, and the alimentary canal.^6^^,^^7^^,^^8^

Insect taste-sensing occurs via the contact of the stimulus with gustatory receptor neurons (GRNs) hosted within the sensillae.^9^ Insect gustatory sensillae commonly occur on the mouthparts, and sometimes on other external appendages such as the ovipositor, antennae, and tarsal and pre-tarsal leg segments.^10^ In addition, sensory cells located in internal tissues (e.g., gut, fat body, and brain) detect nutrients and metabolically processed derivatives that mediate energy homeostasis and modulate feeding behaviors.^11^ The molecular basis for sugar detection in insects is best characterized in *Drosophila melanogaster* and mainly involves the Gr5/61/64 clade composed of eight phylogenetically related sugar gustatory receptors (GRs) (Gr5a, Gr61a, and Gr64a-f) and the distantly related GR43a clade.^12^^,^^13^ The Gr5/61/64 clade includes two lineages.^12^ The Gr5a lineage (Gr5a, Gr61a, Gr64b, Gr64e, and Gr64f) responds to trehalose, glucose, and melezitose, while the Gr64a lineage (Gr64a, Gr64c, and Gr64d) responds to sucrose, maltose, maltotriose, and fructose.^14^^,^^15^ Members of the GR43a clade, including lepidopteran GR9s, specifically respond to D-fructose in *D. melanogaster*,^11^ *Bombyx mori*,^16^ *Plutella xylostella*,^17^ and *Apis mellifera.*^18^ By contrast, the *Helicoverpa armigera* GR9 exhibits a broader sugar response profile.^19^ In the *D. melanogaster* brain, GR43a acts as a narrowly tuned fructose sensor that promotes feeding in hungry flies but suppresses feeding in satiated flies.^11^ In the midgut, GR43a-expressing neurons detect dietary fructose to regulate the expression and secretion of carbohydrate-digesting enzymes and modulate peristaltic movements, which promote efficient digestion, absorption, and secretion.^20^ In general, the functional characterizations of insect sugar GRs indicate that they are activated by millimolar sugar concentrations.^14^^,^^16^

**Figure 1:**
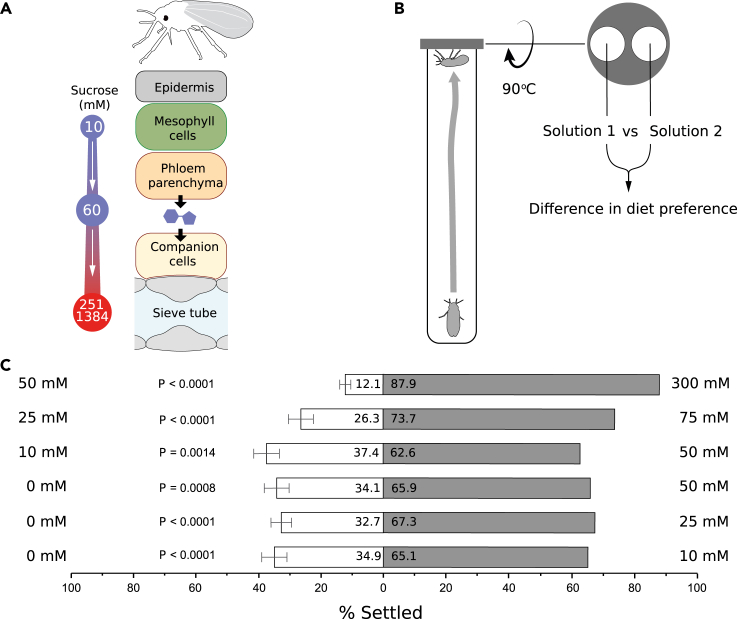
*B. tabaci* adults can sense, discriminate, and choose between different concentrations of sucrose (A) The *B. tabaci* stylets encounter increasing apoplastic sucrose concentrations^33^^,^^34^^,^^35^ on their way to the phloem sieve elements located deep within the leaf. Sucrose concentrations in the phloem sap are from Hayashi and Chino and Fink et al.^36^^,^^37^ (B) The dual-choice assay consists of two feeding sites in the lid that subject adults of *B. tabaci* to two diets. (C) The percentage (mean ± SEM) of *B. tabaci* adults choosing a one diet over the other (68 mM of an amino acid cocktail mimicking the *Arabidopsis thaliana* phloem sap, H_2_O, changing concentrations of sucrose) after 24 h (n > 30 in all experiments, details on the statistical analysis are provided in the ‘STAR Methods’ section).

The host plants of phloem feeders use three main mechanisms for loading sucrose into the phloem.^21^ In most herbaceous plants, including most crop plants, sucrose is loaded into the phloem by an active apoplastic process^22^ in which sucrose synthesized in mesophyll cells moves through plasmodesmata only up to the phloem parenchyma cells (Figure 1A). Then, sucrose is exported across the phloem-parenchyma plasma membrane into the phloem apoplast by SWEET transporters (Sugars Will Eventually be Exported Transporters).^23^ Subsequently, sucrose is imported across the plasma membrane of the companion cells by sucrose transporters and moves through plasmodesmata into the sieve elements.^24^ The symplasmic loading pathway can occur in an active or passive manner. Many trees use passive symplasmic loading, in which the entire route from mesophyll cells to the phloem sieve elements is connected by plasmodesmata, and sucrose moves down a concentration gradient until the phloem sieve tube.^25^ A limited number of herbaceous plants and trees use active (polymer-trap) symplasmic loading. In this case, sucrose passes from bundle sheath cells into specialized companion cells through specialized plasmodesmata and converted into trisaccharides (like raffinose) and tetrasaccharides (like stachyose) before entering the sieve elements for long-distance transport.^26^ The polymerization reduces sucrose concentration in the sieve tube, which facilitates greater transport from the mesophyll to the phloem due to increased concentration gradient.^27^ It has been shown that some polymer-trapping loading species employ both polymer-trapping and apoplastic loading mechanism.^28^ Moreover, even in long-distance transport of sucrose via the passive symplastic loading pathway, a small portion of sucrose may leak into the apoplast outside of the sieve element–companion cell complexes.^29^ Importantly, both polymer-trap loaders and plants producing sugar alcohols translocate significant amount of sucrose in parallel to the aforementioned special sugars.^30^^,^^31^^,^^32^

Although research on phloem feeding has been conducted for more than one hundred years, a surprisingly obvious question hinders our ability to understand this specialized feeding mode. How do phloem feeders find their feeding site, the phloem sieve elements (Figure 1A)? Phloem feeders should be able to locate sieve elements through sensing of sucrose or other chemical gradients from the plant surface to the vascular bundle, but no unequivocal proof for this has been provided so far, nor a putative molecular mechanism/s have been discovered.^38^^,^^39^ The aforementioned cited studies focused on aphids, suggesting a “rejection-acceptance” model that involves an intercellular pre-programmed stylet progression toward the sieve elements with regular intracellular sampling punctures along the pathway, until the “right” sucrose concentration and pH are detected. We believe that this model is a good starting point, but argue that it holds a few gaps, and lacks mechanistic details that prevent its universal usage for phloem feeders such as whiteflies that hardly make extra-phloem intracellular punctures.^40^

We focused here on the whitefly *Bemisia tabaci*, a hemipteran phloem feeder belonging to the sternorrhynchan suborder. *B. tabaci* is considered a delicate phloem feeder, because the pathway of its stylets from the leaf surface, through the apoplast and up to the phloem sieve elements involves only few intracellular punctures, which generally occur only after the stylets have penetrated deep into the leaf tissue.^40^ Furthermore, salivation and ingestion behaviors during intracellular punctures have not been detected^7^ although we cannot exclude the possibility that the turgor pressure of the punctured cells can force sap up to the precibarial chemoreceptors. This suggests that *B. tabaci* (and likely other whiteflies) might not locate their feeding site by frequent sampling of the cells along the pathway like aphids do (see above), but by an alternative yet to be described mechanism.

Using choice assays, we provide evidence that adult *B. tabaci* discriminate between diets containing zero, 10, 25, 50, 75, and 300 mM sucrose, which correspond to sucrose concentrations found in the plant leaf tissues, from the surface, through the mesophyll apoplast and up to the phloem sieve elements.^33^^,^^34^^,^^35^ We hypothesized that sugar GRs play a major role in the ability of the insect to sense differences in sucrose concentrations, and used bioinformatic/phylogenetic analyses to identify four candidate sugar GRs in *B. tabaci*. Using the two-electrode voltage clamp of *Xenopus* oocytes, we show one of them, *Gr1* (*BtabGr1*, NCBI: XP_018911724), to be narrowly tuned to sucrose. Moreover, we report that silencing of *BtabGR1* expression by dsRNA feeding significantly interferes with the ability of *B. tabaci* adults to discriminate between equivalent apoplast and phloem sap concentrations of sucrose. The possible role of BtabGR1 in sensing increasing gradient of apoplastic sucrose concentrations and evaluating gut sucrose concentrations is discussed.

## Results

### *B. tabaci* adults discriminate between different sucrose concentrations

Using a dual-choice bioassay, we examined the ability of *B. tabaci* adults to discriminate between different sucrose concentrations (Figure 1B). We first examined whether *B. tabaci* displays a threshold for sucrose detection by allowing the insects to choose between a diet containing 0 mM sucrose (water) and diets containing low sucrose concentrations (10, 25, and 50 mM). Adult *B. tabaci* demonstrated a moderate but highly significant preference (65.1% [p < 0.0001]; 67.3% [p < 0.0001]; and 65.9% [p = 0.0008]) toward the 10, 25, and 50 mM sucrose solutions, respectively (Figure 1C). Next, we asked whether *B. tabaci* adults choose between two putative apoplastic concentrations of sucrose (10 mM versus 50 mM and 25 mM versus 75 mM). A majority (62.6%) of insects significantly preferred a 50 mM over a 10 mM sucrose diet solution and a larger proportion of adults (73.7%) significantly preferred a 75 mM over a 25 mM sucrose solution (p = 0.0014 and p < 0.0001, respectively) (Figure 1C). Finally, we tested if adult *B. tabaci* choose between an apoplastic and phloem sap concentrations of sucrose. A large majority of adults (87.9%) significantly preferred a 300 mM over a 50 mM sucrose solution (p < 0.0001) (Figure 1C). In all tests where the two chambers had identical solutions, there were no significant differences in preference (p ≥ 0.185, Table S1), indicating that significant differences between different solutions (Figure 1C) were not the result of an artifact in our experimental system. Taken together, these findings not only suggest that *B. tabaci* adults can detect low apoplastic concentrations of sucrose (10 mM) but also raise the possibility that these insects can follow an increasing sucrose gradient found in the apoplast and to recognize their feeding site, the phloem sieve elements.

### The *B. tabaci* genome contains four sweet gustatory receptor candidates

**Figure 2:**
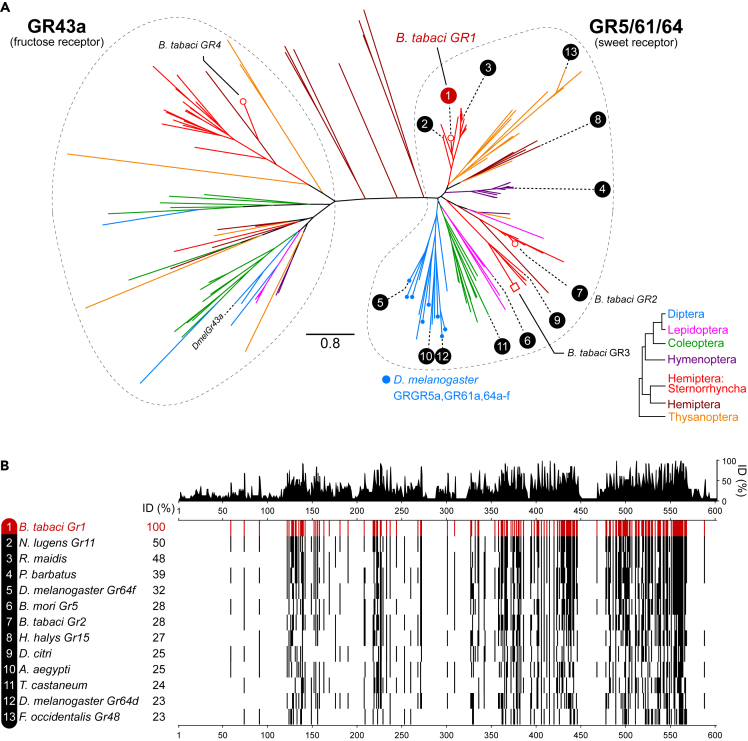
A unique clade of sugar receptors has emerged in sternorrhynchans (A) A phylogenetic tree of insect sugar GRs suggests two main lineages, the GR43a and the Gr5/61/64 groups, and the presence of a distinct clade within the Gr5/61/64 group that harbors only sweet GRs belonging to species from the Sternorrhyncha suborder of Hemiptera. Insect orders are marked by different branch colors. The four identified *B. tabaci* GRs are marked with solid white circles (complete coding sequence) or square (partial coding sequence) shapes. (B) Amino acid sequence alignment of representative sweet GRs from the GR61/64/5 lineages (Figure S3). The indicated pairwise amino acid sequence identity (ID) is to the BtabGR1sequence.

We hypothesized that the observed ability of *B. tabaci* adults to choose between different sucrose concentrations is mediated by sugar GRs. Therefore, we searched the annotation files of the *B. tabaci* genome project^41^ and the NCBI *B. tabaci* genome annotation file (NCBI: ASM185493v1) for the terms “sugar”, “sweet”, and “gustatory” and for GO/Pfam terms associated with sugar/sweet taste sensing. We also searched for *B. tabaci* homologs to all characterized sugar receptors of insects. We found that the *B. tabaci* genome harbors four gene candidates: *BtabGR1* (NCBI: XP_018911724), *BtabGR2* (NCBI: XP_018903763), *BtabGR*3 (NCBI: XP_018914560), and *BtabGR4* (NCBI: XP_018910036). To infer the phylogenetic relationships of these genes, we used several bioinformatic approaches to identify candidate sugar GRs from 41 species belonging to six insect orders and generated a maximum likelihood tree (Figures 2A, S1 and Table S2).

The maximum likelihood tree was found to be composed of two main sub-trees, one containing the sugar GR homologs of the *D. melanogaster* GR5/61/64 clade and the other containing the sweet GR homologs of *D. melanogaster* GR43a. The GR5/61/64 sub-tree includes three *B. tabaci* sugar GRs: *BtabGR1*, *BtabGR2*, and *BtabGR3*. *BtabGR2* and *BtabGR*3 show close phylogenetic relationships to other insect homologs of the GR5/61/64 clade. *BtabGR1*, on the other hand, resides within a distinct clade inside the GR5/61/64 sub-tree enriched in candidate GRs belonging to species from the Sternorrhyncha suborder of Hemiptera. *BtabGR4* was found to be the only sugar GR gene in the *B. tabaci* genome associated with the GR43a sub-tree. It resides within a clade containing only sugar GRs of hemipteran species. Alignment of representatives from different clades of the GR5/61/64 sub-tree revealed that they all share a conserved region in their C-terminus (Figure 2B).

### *BtabGR1* is a sucrose receptor

**Figure 3:**
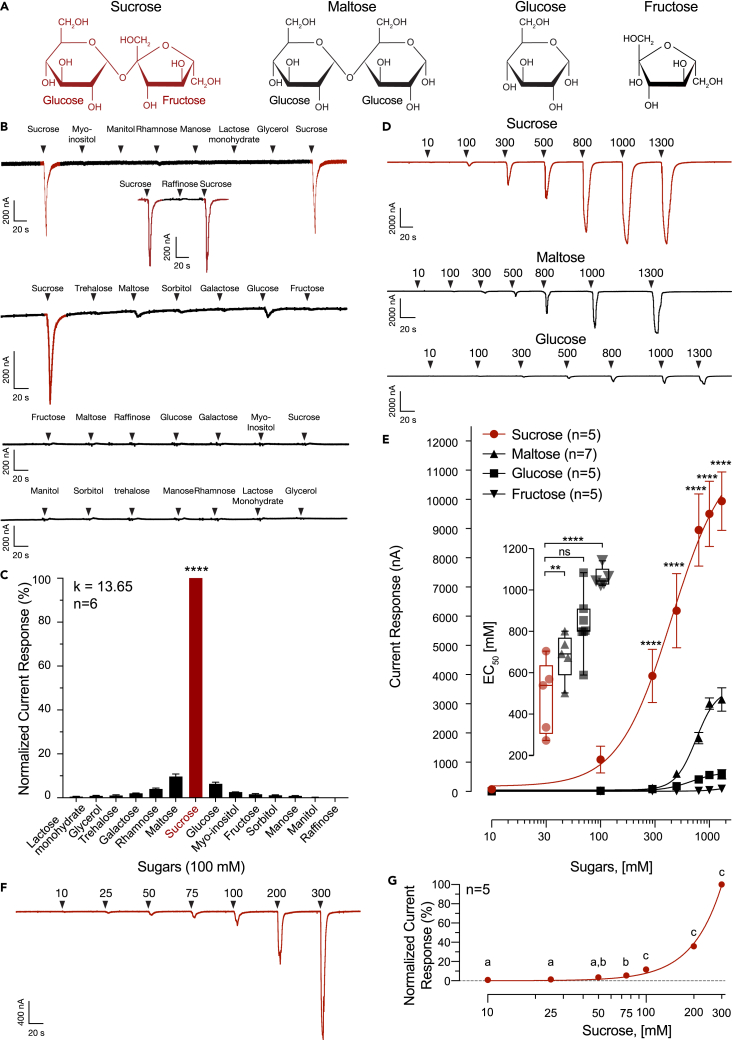
BtabGR1 is a sucrose receptor (A) Haworth projections of sucrose, maltose, glucose, and fructose. Sucrose is composed of glucose and fructose subunits. (B) Representative current traces of BtabGR1 and water-injected *Xenopus* oocytes in response to 100 mM of 14 sugar molecules. Black arrows indicate sugar delivery. (C) BtabGR1 is narrowly tuned to sucrose (kurtosis value = 13.65). The mean current responses (±SEM, n = 6) to 100 mM of 14 sweeteners were normalized to sucrose (one-way ANOVA; ∗∗∗∗p < 0.0001). (D) Representative current traces of BtabGR1 in response to 10-fold serial dilutions of sucrose, maltose, glucose, and fructose. (E) Sucrose was the most efficacious (two-way ANOVA followed by Tukey’s post-hoc test; ∗∗∗∗p < 0.0001) and most potent ligand (one-way ANOVA; ∗∗p = 0.0033, data are represented as mean ± SEM). (F) Representative current trace of BtabGR1 in response to low millimolar sucrose concentrations. (G) Concentration-response relationship between BtabGR1 current responses and low sucrose concentrations (two-way ANOVA followed by Tukey’s post-hoc test).

As *BtabGr1* was found to reside within a distinct clade of the GR5/61/64 sub-tree containing many candidate GRs from the Sternorrhyncha suborder, our next step was to explore its molecular receptive range using a panel of sugar compounds, including sucrose, maltose, glucose, and fructose (Figure 3A). We used the two-electrode voltage clamp of *Xenopus* oocytes to screen the receptor with a panel of 14 carbohydrate molecules (Table S3), including sugars reported to be present in the phloem sap or in other plant tissues. Administration of myo-inositol, mannitol, rhamnose, mannose, lactose monohydrate, glycerol, raffinose, trehalose, sorbitol, galactose, and fructose did not elicit any significant responses (Figure 3B). By contrast, sucrose, maltose, and glucose (Figure 3A) evoked significant current responses (Figure 3B). Current amplitude responses to sucrose were significantly higher than maltose and glucose (Figure 3C). These findings suggest that BtabGR1 is a narrowly tuned sucrose receptor (Kurtosis = 13.64) (Figure 3C).

While fructose did not elicit significant responses in our screen (Figure 3B), its presence in sucrose warranted further functional analysis. To evaluate the relationships between BtabGR1 and sucrose, maltose, glucose, and fructose, we established four concentration-response curves and interpolated their respective EC_50_ values, which were in the millimolar range (Figures 3D and 3E). The activation threshold of BtabGR1 was the lowest for sucrose, which is the sugar that elicited the largest currents at all tested sugar concentrations (Figures 3F and 3G). In terms of current amplitudes, maltose was the second most efficacious ligand followed by glucose and fructose (Figure 3E). The median EC_50_ values of sucrose (539 mM), maltose (803 mM), and fructose (1043 mM) were found to be significantly different. The EC_50_ values between sucrose and glucose (691 mM) were not statistically different (Figure 3E).

In order to link between our behavioral (artificial-diet choice assays) and functional characterizations, we increased the resolution of the concentration-response relationship of BtabGR1 toward lower sucrose concentration range. While 25 and 50 mM sucrose concentrations elicited minute yet visible currents (Figure 3F), they were not statistically different from 10 mM-induced currents (Figure 3G). Sucrose concentrations above the 75 mM threshold evoked significant currents and 100 mM sucrose concentration and above elicited exponential increases in the current (Figure 3G).

### Silencing of *BtabGR1* interferes with the ability of *B. tabaci* to discriminate between sucrose concentrations

**Figure 4:**
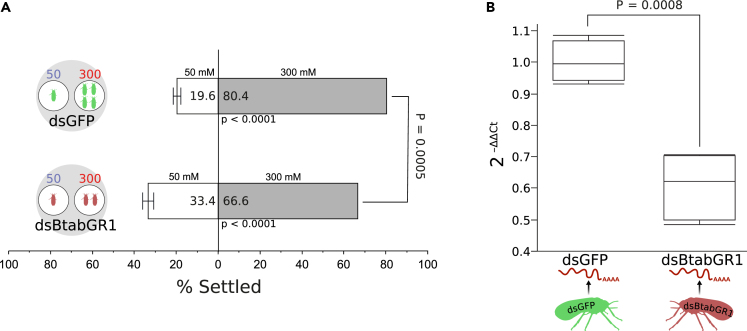
Silencing of *BtabGR1* interferes with the ability to discriminate between sucrose concentrations (A) The percentage (mean ± SEM) of *B. tabaci* adults choosing 300 mM over 50 mM sucrose after 72 h of feeding on *dsBtabGR1* (n = 50) or *dsGFP* (n = 46) containing diets (details on the statistical analysis are provided in the ‘STAR Methods’ section). (B) An RT-PCR assay indicating a significant reduction in the expression (mean $2^{-\Delta \Delta Ct}$ ± SEM) of the *BtabGR1* gene in whole body homogenates of *dsBtabGR1*- versus *dsGFP*-fed insects (p *=* 0.0008, n = 4).

To learn more on the involvement of *BtabGR1* in sucrose sensing, we tested the effect of *BtabGR1* silencing on the ability of *B. tabaci* adult to discriminate between apoplast (50 mM) and phloem sap (300 mM) concentrations of sucrose. Insects feeding for 72 h on both *dsBtabGR1* and *dsGFP* diets were able to discriminate and prefer the 300 mM over the 50 mM diet (p < 0.0001). However, a significant reduction in this ability was observed in adults feeding on the *dsBtabGR1* diet, when compared to adults feeding on *dsGFP* (66.6% versus 80.4%, respectively, p *=* 0.0005) (Figure 4A). An RT-PCR assay conducted to verify the silencing effect indicated a significant reduction of 40% in the expression of the *BtabGR1* gene in whole body homogenates of *dsBtabGR1*- versus *dsGFP*-fed insects (Figure 4B), drawing a direct link between the disrupted feeding behavior and the expression level of the gene. Further PCR analyses also indicated that the *BtabGr1* gene is expressed in body regions/tissues relevant to sucrose sensing in phloem feeders, the head and the abdomen (Figure S2). Taken together, these results suggest that BtabGR1 is likely to play an essential role in the ability of *B. tabaci* to sense sucrose and to discriminate between apoplast and phloem sap concentrations of sucrose.

## Discussion

### BtabGR1 might play a major role in the ability of *B. tabaci* to locate its feeding site

In this paper, we explored the potential sensory mechanism underlying the ability of phloem-feeding insects to locate their feeding site deeply buried within the leaf tissue. We focused our investigation on the phloem-feeding whitefly *B. tabaci*. We present complementing behavioral, molecular evolution, gene expression and functional evidence suggesting that one of the sugar receptors, BtabGR1, is likely to mediate sucrose sensing in *B. tabaci*.

As outlined in a previous section, various concentrations of sucrose are present in the leaf apoplast, the intercellular space used by whiteflies, and other phloem feeders for stylets penetration and navigation toward their feeding site, the phloem sieve elements.^7^ These concentrations can range from 2 to 7 mM^33^ to high concentrations of ∼60 mM in the apoplast of vascular parenchyma cells.^34^^,^^35^ Our behavioral choice assays indicated that *B. tabaci* adults detect, discriminate, and significantly choose between diets containing 0, 10, 25, 50, 75, and 300 mM of sucrose, with preference each time for the higher concentration provided. Still, at sucrose concentrations between 0 and 50 mM, the ability to choose the higher concentration was somewhat weaker (65%–67%) when compared to the ability to choose to feed on 75 versus 25 mM (74%) (marginally significant, p ≤ 0.0966, one-way ANOVA followed by pairwise comparisons to the 0 versus 10, 25, and 50 and 10 versus 50 mM sucrose choice assays) or 300 versus 50 mM (88%) (p ≤ 0.0074, one-way ANOVA followed by pairwise comparisons to the 0 versus 10, 25, and 50, 10 versus 50, and 25 versus 75 mM sucrose choice assays) suggesting the possible involvement of complementing mechanisms such as the sensing of osmotic pressure.^3^ In addition, the pharmacological measurements indicated that the molecular receptive range of BtabGR1 correlates well with both the reported range of sucrose concentrations in different parts of the leaf apoplast and the behavioral choice assays, as 10, 25, and 50 mM sucrose concentrations elicited minute yet visible currents, while sucrose concentrations around a 75 mM threshold and 100 mM evoked significant and exponential current increases, respectively. Moreover, silencing the expression of the *BtabGR1* gene by dsRNA feeding significantly interfered with the ability of *B. tabaci* adults to discriminate between apoplast (50 mM) and phloem sap (300 mM) concentrations of sucrose, pointing to the major role BtabGR1 is likely to play in the insect’s ability to locate its feeding site.

Taking all these data together brings us to propose a new model for the movement of the stylets of *B. tabaci* (and likely other delicate phloem feeders) in the leaf apoplast. The model specifically depends on the ability of the insects to sense an increasing gradient of apoplastic sucrose concentration. In agreement with Hewer et al.,^38^^,^^39^ we propose initial stylet penetration is behaviorally programmed to occur perpendicular to the plant surface through apoplast regions that are far from the vascular bundle (for example close to the leaf surface) as the sucrose concentrations there are low and up to 10 mM.^42^ However, we propose that the movement should become directional at the apoplast of deeper mesophyll cell layers that are close to the phloem parenchyma, due to apoplastic sucrose loading by SWEET transporters^23^ that create a diffusing gradient of sucrose, or the leakage of sucrose into the apoplast from the sieve element–companion cell complexes in symplastic loading systems.^29^ At this stage, the apoplastic fluids are likely to move from the stylet tips to the precibarial GRs using capillary forces which build liquid bridges (i.e., bridges that are formed when liquid is placed between two hydrophobic surfaces due to the surface tension of the liquid).^43^^,^^44^ Interestingly, our phylogenetic analysis placed BtabGR1 within a clade enriched in Sternorrhyncha species, raising the possibility that a specialized phloem-feeding habit might have shaped the molecular receptive range of this receptor to become the most “narrowly tuned” sucrose receptor characterized up to date. In this respect, we posit that the weak responses to maltose, glucose, and fructose are remnants of broader ancestral activity. This sensitivity may not have significant biological importance as the concentrations of all three sugars in the phloem sap and whole leaf are in the 0 (not detectable) – 15 mM range,^2^ which is well below the observed BtabGR1 sensitivity to these sugars.

### The putative expression sites of BtabGR1

Our analyses so far indicated that *BtabGR1* is expressed both in head and abdomen tissue samples of the whitefly *B. tabaci*. In the head, *BtabGR1* may be expressed in the precibarium and cibarium chemosensory sensillae, which serve as the primary peripheral taste organs of phloem feeders.^6^^,^^7^^,^^8^ The BtabGR1 receptor might also be involved in monitoring sugar (sucrose) content of the food in the “filter chamber”, a midgut loop present in most phloem feeders, which allows the shifting of excess water and/or sugar directly to the hindgut for excretion, before absorption of ingestion nutrients takes place in the midgut.^45^ Monitoring the sugar content in the “filter chamber” or the midgut may be carried out by sugar-sensing neurons that send their dendritic terminals into the lumen, a function previously described for the proventricular ganglion of *D. melanogaster*^13^ or by sugar receptors expressed in gut epithelial cells.^46^ The detection of sugars by these receptors can control not only food intake but also induce digestive processes, such as peristaltic movements, activation of metabolic enzymes, and sugar transport. On the other hand, it is less likely that *BtabGR1* is expressed in the sub-esophageal ganglion or other brain neurons, as sugar receptors that are expressed in these neuronal tissues are likely to play a role in monitoring the levels of glucose, fructose, trehalose, or other nutritious sugars in the hemolymph.^11^ More detailed cytological studies will help reveal the cellular location of *BtabGR1* expression and its physiological role in sucrose-sensing in the head and gut tissues.

### Insect GRs are related to insect odorant receptors but can operate without co-receptors

Insect GRs share a common ancestral origin with the insect odorant receptors (ORs).^47^^,^^48^^,^^49^^,^^50^ Insect ORs present a conserved region located in the C-terminus of the proteins, which is thought to be involved with the pore and anchor domain, indicating heteromerization with their obligatory co-receptor (Orco).^51^^,^^52^^,^^53^^,^^54^ Here, we demonstrate that like the ORs, sweet GRs share a very similar pattern of high conservancy in their C-terminus. In contrast to OR-Orco complexes, ligand-sensing GR subunits can operate without co-receptors^14^ but may form higher-order complex comprised of multiple subunits.^53^^,^^54^ The co-expression of more than one GR in the same cell may also modulate sugar selectivity in GR neurons.^55^ For example, the expression of *A. mellifera* sweet receptor *GR1* alone (*AmGR1*) results in robust responses to sucrose, glucose, maltose, and trehalose in a dose-dependent manner while the expression of *AmGr2* alone does not show any response to sugar substances. However, co-expression of these two receptors results in higher sensitivity to glucose and lower sensitivity to sucrose, trehalose, and maltose.^56^ Coincidentally, the individual expression of two sugar receptor genes (*TchiGR64f1* or *TchiGR64f2*) of the parasitic wasp *Trichogramma chilonis* did not elicit any responses to 11 tested sugars while their co-expression produced distinct responses to sucrose.^57^ These findings suggest that inter-subunit interactions between GR subunits (homomeric or heteromeric) may form receptor complexes with distinct sugar selectivities.^53^

### Conclusion

Our study is a first step in a long journey to understand the molecular and physiological underpinnings of sugar perception in phloem-feeding insects. Here, we have focused our research on the phloem-feeding whitefly *B. tabaci*. Contrary to humans, who use one sweet receptor broadly tuned to a variety of sweet molecules, *B. tabaci* has evolved at least four candidate sugar receptors of which BtabGR1 exhibits narrowly tuned selectivity toward sucrose, the dominant sugar component in their diet. Our analyses indicate that BtabGR1 responds to sucrose concentrations found in the apoplast surrounding the phloem parenchyma, suggesting a role in feeding site location. Moreover, *BtabGR1* expression in the abdomen suggests an additional/complementing role in gastrointestinal sucrose sensing. Future molecular, cytogenetic, and tissue transcriptomics approaches will help tease out if these two putative activities are mutually exclusive. Targeting this novel receptor and understanding its physiological roles may also provide new tools for controlling the global impact *B. tabaci* exerts on agriculture.

### Limitation of the study

Further studies are required to determine the response profiles of the BtabGR2, BtabGR3, and BtabGR4 receptors to different sugars. For example, these receptors might be involved in sensing the levels of glucose, fructose, trehalose, or other nutritious sugars in the hemolymph for determining the insect’s satiety state. We do not think that BtabGR2, BtabGR3, and BtabGR4 play a significant role in feeding site location because our silencing assays indicated that BtabGR1 is the main (if not solely) sucrose receptor (the ability to discriminate between 50 and 300 mM sucrose was significantly reduced in silenced insects). In addition, at this stage, we cannot completely exclude the possibility that silencing of BtabGR1 has other non-direct effects on the insect’s behavior, although we find this possibility unlikely as we did not observe any reduced performance in our dual-choice experimental system.

## STAR★Methods

### Key resources table

**Table:** 

REAGENT or RESOURCE	SOURCE	IDENTIFIER
**Chemicals, peptides, and recombinant proteins**		

AA mixtures for artificial diets	Wilkinson and Douglas,^58^	Table S6

**Deposited data**		

Phylogeny protein sequence alignment	This paper; Mendeley Data	https://doi.org/10.17632/9vmnzdww2v.1
Raw choice assays results	This paper; Mendeley Data	https://doi.org/10.17632/9vmnzdww2v.1
Raw electrophysiology results	This paper; Mendeley Data	https://doi.org/10.17632/9vmnzdww2v.1

**Experimental models: Organisms/strains**		

*Bemisia tabaci*	In house population	N/A
pigmented *Xenopus* laevis	Nasco (Fort Atkinson, WI, USA)	N/A

**Oligonucleotides**		

Primers for amplification of several *BtabGr1* regions	This paper	Table S5
Primers for qRT-PCR	This paper	Table S5

**Software and algorithms**		

interproscan v5.47-82.0	Jones et al.^59^	https://interproscan-docs.readthedocs.io/en/latest/HowToRun.html
Augustus v3.3.3	Stanke et al.^60^	https://github.com/Gaius-Augustus/Augustus
Orthofinder v2.2.7	Emms and Kelly,^61^	https://github.com/davidemms/OrthoFinder
JMP Pro 16.0	SAS Institute, Cary, NC	https://www.jmp.com/
CD-HIT v4.6	Fu et al.^62^	https://sites.google.com/view/cd-hit
MAFFT v7.215	Katoh and Standley,^63^	https://mafft.cbrc.jp/alignment/software/
Gblocks v0.91b	Castresana,^64^	https://home.cc.umanitoba.ca/∼psgendb/doc/Castresana/Gblocks_documentation.html
IQ-TREE v1.6.5	Nguyen et al.; Kalyaanamoorthy et al.^65^^,^^66^	http://www.iqtree.org/

**Other**		

*Xenopus* irradiated diet	Zeigler, Gardners, PA, USA,	Prod. No. 316518-18-2412

### Resource availability

#### Lead contact

Further information and requests for resources and reagents should be directed to and will be fulfilled by the lead contact, Ofer Aidlin Harari (ofer.aidlinhara@mail.huji.ac.il).

#### Materials availability

This study did not generate new unique reagents.

### Experimental model and subject details

#### *Bemisia tabaci* rearing and collecting protocols

A population of the local (Israeli) species of the whitefly *B. tabaci* (the MEAM1 species) was used. The population was established in 2019 by collecting ∼ 6,000 adults from four tomato and watermelon open-fields. Since its collection, the population has been maintained on both host plants (six cages contributing equally to each new generation) in the laboratory insectaries (28 ± 2°C and a 14:10-hr light:dark cycle). All choice assays were conducted on a sub-population of the one described above reared for more than 20 generations only on cotton plants.

The whiteflies used in the choice assays were randomly assigned newly emerged adults (∼3 days-old), this age was found to be optimal for behavior assays. As couples (male and female) were collected, male to female sex ratio is ∼1:1.

#### *Xenopus laevis* rearing

The *Xenopus laevis* females (pigmented *Xenopus laevis*) were obtained from Nasco (Fort Atkinson, WI, USA). The frogs were reared in a XenoPlus amphibia housing system (Tenciplast, West Chester, PA, USA) under controlled water conditions (pH = 7.6, Temp = 18.2°C, conductivity = 1100 μS). The frogs were fed three time a week with adult *Xenopus* irradiated diet (Zeigler, Gardners, PA, USA, Prod. No. 316518-18-2412).

All applicable international, national, and/or institutional guidelines for the care and use of animals were followed (NIH approval number: OPRR-A01-5011).

### Method details

#### Dual choice assay

In our custom-designed dual choice-chamber system (30 x 200 mm glass vials), sucrose solutions were placed between two layers of Parafilm (4 x 4 cm) stretched over a 3D-printed lid with two open round windows (Figure 1B). Each sucrose solution was supplemented with 68 mM of an amino-acid cocktail chemically mimicking the *Arabidopsis thaliana* phloem-sap^58^ (Table S6). We assumed that the Parafilm is perceived by the insects as the plant cuticle.^39^ Moreover, we considered the subsequent steps in which stylet penetration of the Parafilm, probing and sampling take place, as a mimic of apoplast and/or sieve-element evaluation. Therefore, a decision to leave the test solution or to continue ingesting can be interpreted as rejection or acceptance of specific nutritional conditions in plants.

Eighty newly emerged adults (∼3 days-old) were collected into a glass vial using a keyboard vacuum cleaner. Next, the two-way choice lids containing two feeding sites (Figure 1B) filled with treatment solutions, were placed on top of the vial, and sealed together using a parafilm strip. Each vial was covered with aluminum foil leaving only the lid exposed to light. After 24 h (at 28°C and 14:10 L:D photoperiod), the number of settled adults in each of the two feeding sites was recorded. To control for potential position effect, we rotated by 180º the lids of the replicates so that the position of the two feeding sites was alternated between replicates. Approximately thirty replicates were carried out for each treatment. The following set of choice assays were conducted: 0 mM versus 10 mM of sucrose, 0 mM versus 25m M of sucrose, 0 mM versus 50 mM of sucrose, 10 mM versus 50 mM of sucrose, 25 mM versus 75 mM of sucrose, and 50 mM versus 300 mM of sucrose. To exclude the possibility that factors other than differences in sucrose concentrations or position effects were affecting the insects’ preference, relevant choice assays presenting identical concentrations of sucrose in the dual choice-chamber were conducted simultaneously: 10 mM versus 10 mM, 25mM versus 25mM, 50 mM versus 50 mM, 75 mM versus 75 mM and 300 mM versus 300 mM of sucrose. These assays used the same cohort of adult whiteflies and the same experimental conditions. Adults were assigned randomly to the dual choice-assays containing either different or identical sucrose concentrations in the two feeding sites. The 0 mM versus 0 mM sucrose assays were not conducted as the adults did not survive the 24 h choice period.

#### Sugar *GRs* identification and phylogenetic analysis

Insect GR sequences were collected using the following procedures: (I) manual inspection of insect genomic/transcriptomic data (published in NCBI) for the presence of sugar GR-associated GO/Pfam terms (GO0050916, GO003304, GO1903767, GO0090683, GO0033041 and PF06151). Published annotations were used when available with the addition of *in-house* annotations of predicted proteins using interproscan v5.47-82.0 (default parameters, Pfam database 33.1).^59^ The GR proteins of *Phenacoccus solenopsis* were predicted from the published genome (GCA_00961765) using Augustus v3.3.3.^60^ (II) The identification of homologs using *D. melanogaster* sugar GRs (obtained searching “gustatory receptor” in https://flybase.org/) and other functionally characterized insect sugar GRs as queries (for details and references see Table S4) using ‘Orthofinder’ v2.2.7^61^ (default paremeters) and ‘get-homologues’ (default paremeters).^67^ (III) BLASTP search in NCBI using the XP_018911724 sequence as query. This provided 100 best hits that were included in our analysis. (IV) Other sugar GR sequences from published literature.^68^^,^^69^^,^^70^^,^^71^^,^^72^^,^^73^ The combined sugar GR database was screened for duplications and allelic variants using CD-HIT v4.6^62^ with a cut-off of 98% identity. The remaining 184 sequences were aligned using MAFFT v7.215^63^ (default paremeters) and blocks of phylogenetically informative positions were chosen using Gblocks v0.91b (most permisive paremeters per alignment).^64^ At the first step, a general GR tree was built using the Maximum-Likelihood method implemented in IQ-TREE. The amino-acid evolution model was chosen by IQ-TREE prior to tree building (v1.6.5, 1000 replicates SH-aLRT, 5000 ultrafast bootstraps).^65^^,^^66^ At the second step, only the GR sequences that clustered within the sugar receptor clades and selected non-sugar GR outgroups were used to produce a Maximum-likelihood tree of sugar GRs using MAFFT v7.215, Gblocks v0.91b, and IQ-TREE v1.6.5. Pairwise alignments and percent identity calculations were conducted using MAFFT v7.215 and UGENE v1.31.1.^74^

#### *BtabGr1* cloning and mRNA expression in *Xenopus laevis* oocytes

*BtabGr1* cDNA was cloned into pSP64T (Addgene, CAT# 15030, Watertown, MA, USA), using Takara-Clontech infusion kit (CAT# 638909). *In-vitro* transcription was done using the mMESSAGE Mmachine sp6 Transcription kit (CAT# AM1340, Thermo Fisher, Waltham, MA, USA). Stage IV-VI oocytes of *Xenopus laevis* were harvested, mechanically disrupted by hand, and treated with a 10 mg/ml collagenase solution (Sigma Aldrich, CAS) at 18°C for 25 minutes at 60 RPM. Following collagenase treatment, oocytes were washed 5 times in ND96 solution (96 mM NaCl, 2 mM KCl, 5 mM MgCl_2_, and 5 mM HEPES, 1L double-distilled water, pH 7.6). Subsequently, oocytes were rinsed in a washing solution (ND96 solution supplemented with 100 μg/mL gentamicin). Finally, oocytes were washed 5 times with incubation medium (ND96 solution supplemented with 0.8 mM CaCl_2_, 5% dialyzed horse serum, 50 μg/mL tetracycline, 100 μg/mL streptomycin and 550 μg/mL sodium pyruvate). Oocytes were allowed to recover overnight prior to injection with 27.6 nL cRNAs (3 μg/μl), incubated at 18°C for 3–4 days in 24 well-plates in 1 ml incubation medium.

#### Pharmacology

Whole-cell currents were monitored and recorded using the two-electrode voltage clamp (TEVC) technique. Holding potential was maintained at -80 mV using an OC725C oocyte clamp (Warner Instruments, LLC, Hamden, CT, USA). Oocytes were placed in a RC-3Z oocyte recording chamber (Warner Instruments, LLC, Hamden, CT, USA) and exposed to three second-long stimuli. All compounds were solubilized in 200 μL of DMSO prior to dilutions in ND96 buffer supplemented with 0.8 mM CaCl_2_. Data acquisition was carried out with a Digidata 1550A and pCLAMP10 (Molecular Devices, Sunnyvale, CA, USA).

#### dsRNA design, choice assays after gene silencing and qRT-PCR/PCR analyses

The dsRNA constructed against a 370 nt fragment of the green fluorescent protein (*GFP*) gene of the jellyfish *Aequorea victoria* was described in Luo et al.^75^ For designing the dsRNA that targets *BtabGR1*, we first used the gene’s coding sequence as input for DSIR.^76^ Highly ranked siRNAs were aligned to the *BtabGR1* coding sequence and a region (501nt) containing high abundance of mapped siRNAs was identified. Next, all possible 21-mers of the selected region were extracted using Splitter (splitter-size 21 -overlap 20)^77^ and Usearch v.11 (usearch -search_oligodb insect - dbfile -strand both)^78^ was used to verify that all of them do not have exact match or one mismatch to off-target genes in the MEAM1 genome (using the RefSeq annotated genome, GCF_001854935.1, in NCBI).

Choice assays combined with gene silencing were conducted as following. One hundred newly-emerged adults were fed in no-choice glass vials on a solution containing 500 mM sucrose, the aforementioned amino-acid cocktail and 0.5 μg/μl of double stranded RNA (*dsBtabGR1* or *dsGFP* as control). After 48 hours, the lids were replaced by dual choice-assay ones containing again the same amino-acid cocktail and 0.5 μg/μl of *dsRNA* (*dsBtabGR1* or *dsGFP*), and 50 mM versus 300 mM of sucrose. Control for position effect was performed as described above.

For quantification of the silencing effect on *BtabGR1* expression, one-hundred and fifty newly emerged adults were fed for three days on a solution containing 500 mM sucrose, the aforementioned amino-acid cocktail and 0.5 μg/μl of double stranded RNA (*dsBtabGR1* or *dsGFP*). RNA was extracted from the survivors, ∼60 individuals per sample, using the Isolate II RNA mini kit (Meridian Bioscience, Ohio). RNA quality and quantity were evaluated using a NanoDrop 2000 spectrophotometer (Thermo Fisher Scientific, Massachusetts). cDNA was synthesized using 500ng RNA from each sample and the Verso cDNA synthesis kit with Oligo-dT primer (Thermo Fisher Scientific, Massachusetts). The expression level of *BtabGR1* in *dsBtabGR1*- or *dsGFP*-fed adults was examined using the CFX Connect Real-Time PCR System (BIO-RAD, California). A set of primers designed for each target gene (primers 1-4, Table S5). The *B. tabaci* endogenous ribosomal protein L13a (RPL13A) was used as a reference gene.^79^ The qRT-PCR conditions were adjusted for the amplification efficiencies of the target and endogenous genes to be in the log range of 1.9–2.1 and were optimally set to a master mix containing 5μl iTaq Universal SYBR Green Supermix (BIO-RAD, California), 0.5μl of both forward and reverse primers (2pmol/μl), 2μl DDW and 2μl cDNA template. qRT-PCR thermal conditions consisted of 95°C for 2min, followed by 40 cycles of 95°C for 5 sec and 60°C for 30 sec, and an ending cycle of 95°C for 5 sec, 65°C for 5 sec, and 95°C for 30 sec.

For testing the expression of *BtabGr1* in head and gut samples, one hundred heads and fifty abdomens per sample were isolated by dissecting newly emerged adults. RNA extraction and cDNA synthesis with 150ng RNA were conducted as described above. The PCR reaction mixture contained 1μl cDNA template, 1μl of each primer at 10μM (primers 5 & 6, Table S5), 12.5μl PCRBIO-HS mix (PCR Biosystems Ltd., United Kingdom), and 9.5μl DDW. PCR thermal conditions consisted of 95°C for 1.5 min, followed by 40 cycles of 95°C for 15 sec, 60°C for 15 sec, and 72°C for 7 sec, and an ending step of 72°C for 1 min.

### Quantification and statistical analysis

#### Dual choice assay

Differences in diet preference (between the diets offered in the two feeding sites of the chambers) were tested separately for each choice assay using a paired t-test and a null hypothesis of equal distribution (50% of the individuals feeding at each site). When identical sucrose concentrations were offered in the two feeding sites, the proportion of individuals at one of the two feeding sites was used for analysis. The proportional data were arcsin-square root transformed prior to hypothesis testing. As multiple tests were conducted, a false discovery rate (FDR) correction was applied. Statistical significance was assumed at *P* ≤ 0.05. The significance of the differences in diet preference (the proportion of settled adults on the higher sucrose solution) between the 0 mM versus 10 mM, 25 mM and 50 mM, 10 mM versus 50 mM, 25 mM versus 75 mM and 50 mM versus 300 mM sucrose choice assays was analyzed using a one-way ANOVA model followed by *a priori* pairwise comparisons. Prior to the analysis, the proportional data were arcsin-square root transformed. Statistical significance was assumed at *P* ≤ 0.05. All described statistical analyses were conducted using JMP Pro 16.0 (SAS Institute, Cary, NC).

#### Pharmacology

To assess the selectivity of BtabGR1, we used 100 mM ligand solutions each containing one of 13 natural sugars or glycerol (Table S3). Currents were allowed to return to baseline between ligand applications. Our screening protocol included administration of the sugars in forward and reverse orders to control for position effects. Response value of each sugar was normalized to sucrose. The tuning curve and kurtosis value were established using Microsoft Excel (Microsoft Corporation, Redmond, WA, USA).

The four most potent sugars, sucrose, maltose, fructose and glucose were used to establish concentration-response relationships. Seven sugar concentrations were administered for three seconds each and whole cell currents were allowed to return to baseline. The resulting concentration-response curves, EC_50_ interpolation, and statistical analyses were conducted using GraphPad Prism 8 (GraphPad Software Inc., La Jolla, CA, USA).

#### dsRNA design, choice assays after gene silencing and qRT-PCR/PCR analyses

Differences in diet preference (50 mM versus 300 mM of sucrose) were tested separately for each choice assay (diet containing *dsBtabGR1* or *dsGFP*) using again a paired t-test and a null hypothesis of equal distribution (50% of the individuals feeding at each site) (see details above). The paired comparison tests were done simultaneously and with randomly selected whiteflies. The significance of the differences in diet preference (the proportion of settled adults on the 300 mM sucrose diet solution) between the *dsBtabGR1* and *dsGFP* treatments was analyzed using a one-way ANOVA model. Prior to the analysis, the proportional data were arcsin-square root transformed. Statistical significance was assumed at *P* ≤ 0.05.

Quantification of the transcript's expression levels was conducted according to the ΔΔCt method (Applied Biosystems). A one-way ANOVA model determined the significance of the differences between the means of the treatment and control samples (*P* ≤ 0.05).

## Data Availability

• Raw data files from the electrophysiological and behavioral assays and text files of multiple sequence alignments are deposited in Mendeley Data archive and publicly available at https://doi.org/10.17632/9vmnzdww2v.1. • This paper does not report original code. • This paper does not present new sequencing or omics datasets. • Any additional information required to reanalyze the data reported in this paper is available from the lead contact upon request.
