# Supplementary material for: A sucrose-specific receptor in Bemisia tabaci and its putative role in phloem feeding
Source: iScience. 2023 Apr 29;26(5):106752. doi: 10.1016/j.isci.2023.106752 (PMC10206433; doi:10.1016/j.isci.2023.106752)
Supplement: Document S1. Figures S1–S3 and Tables S1–S6 [file mmc1.pdf]

iScience, Volume *26*

Supplemental information

A sucrose-specific receptor in *Bemisia tabaci*

and its putative role in phloem feeding

Ofer Aidlin Harari, Amir Dekel, Dor Wintraube, Yuri Vainer, Rita Mozes-Koch, Esther Yakir, Osnat Malka, Shai Morin, and Jonathan D. Bohbot

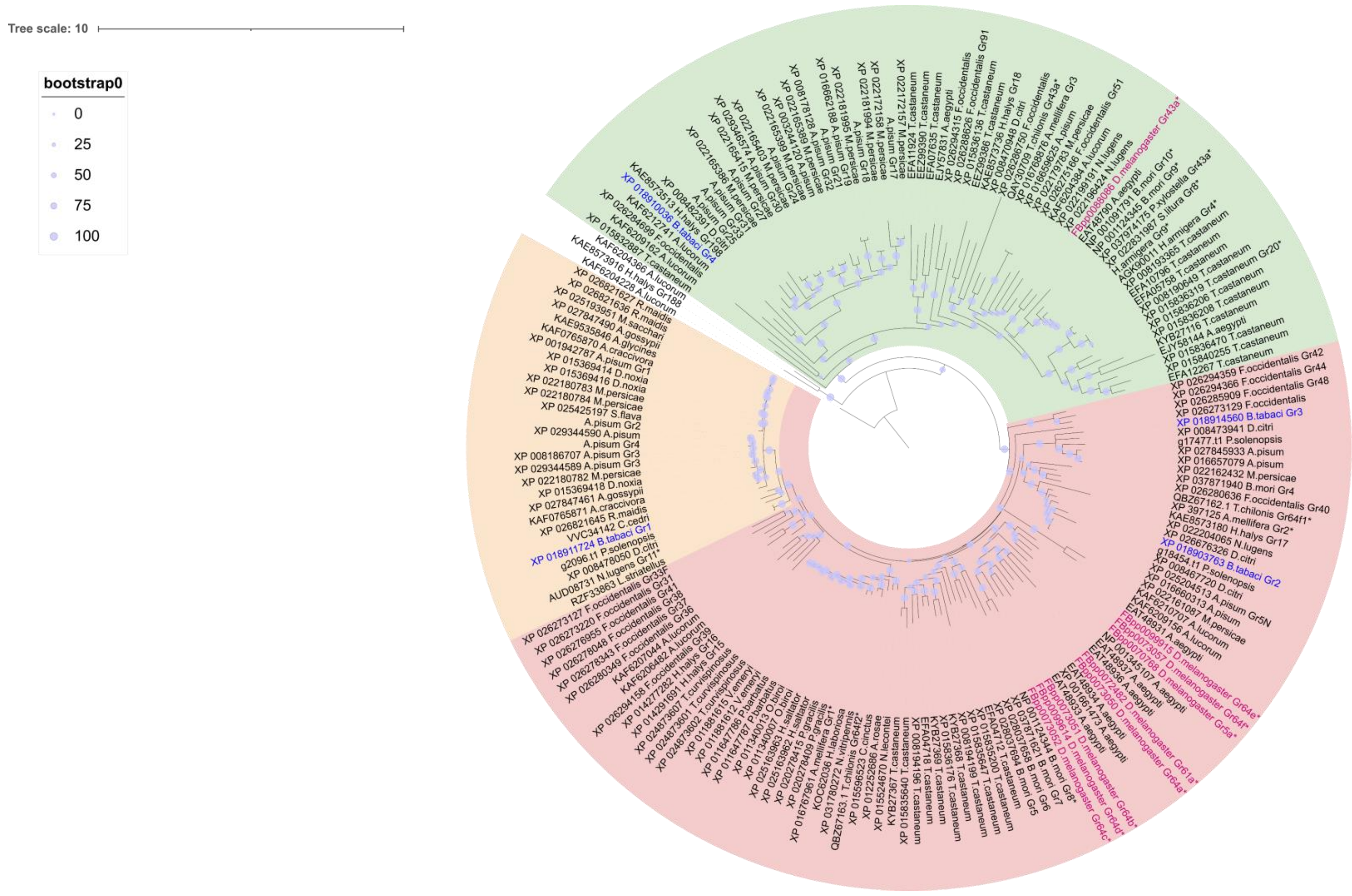

Figure S1. Detailed phylogenetic tree, a detailed version of Figure 2A. Maximum-likelihood tree (based on amino-acid sequences) of insect sugar gustatory receptors (GRs), representing 52 species from seven insect orders. BtabGR1 belongs to a clade within the GR61/64/5 sub-tree enriched in candidate GRs belonging to species from the Sternorrhyncha suborder of Hemiptera. Highlighted taxa: blue, *B. tabaci* GRs; purple, *D. melanogaster* GRs. Highlighted clades: green, DmGR43a sub-tree; red, GR61/64/5 sub-tree; orange, Sternorrhynca enriched clade harboring BtabGR1. Node labels represent bootstrap support (1,000 replicates SH-aLRT support (%) and 5000 ultrafast bootstrap support (%)).

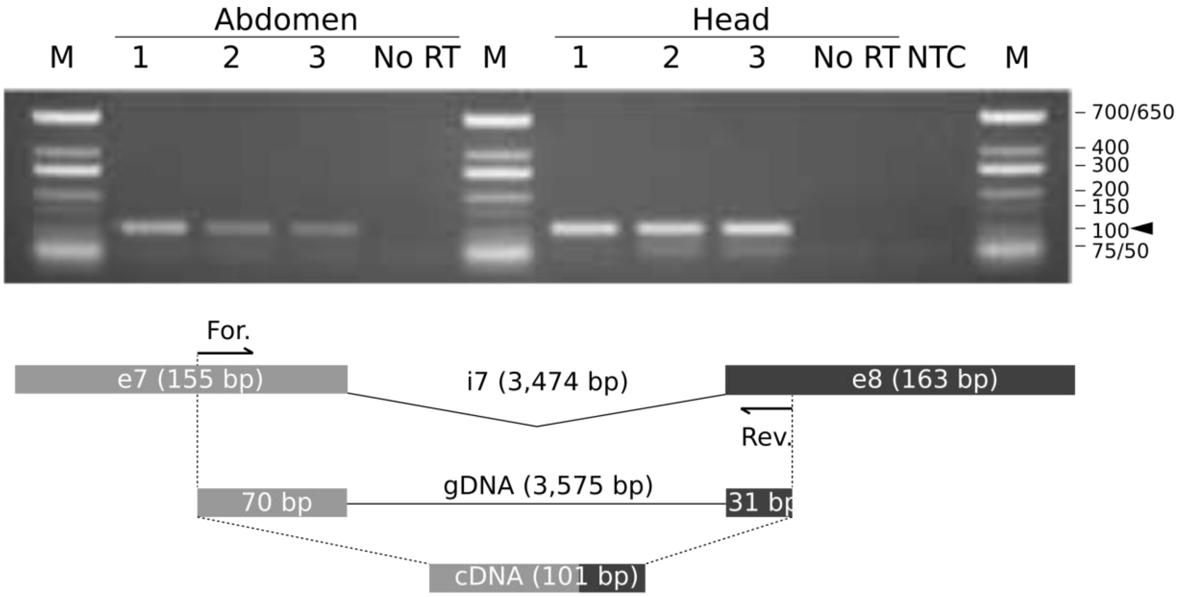

Figure S2. *BtabGR1* is expressed in the head and abdomen of *B. tabaci*, related to Figure 4B. Synthesized cDNAs and genomic DNA (gDNA) from adult head and abdomen tissues of *B. tabaci* were amplified by PCR using primers spanning intron 7 (i7). PCR amplicons were visualized on a 1% agarose gel.

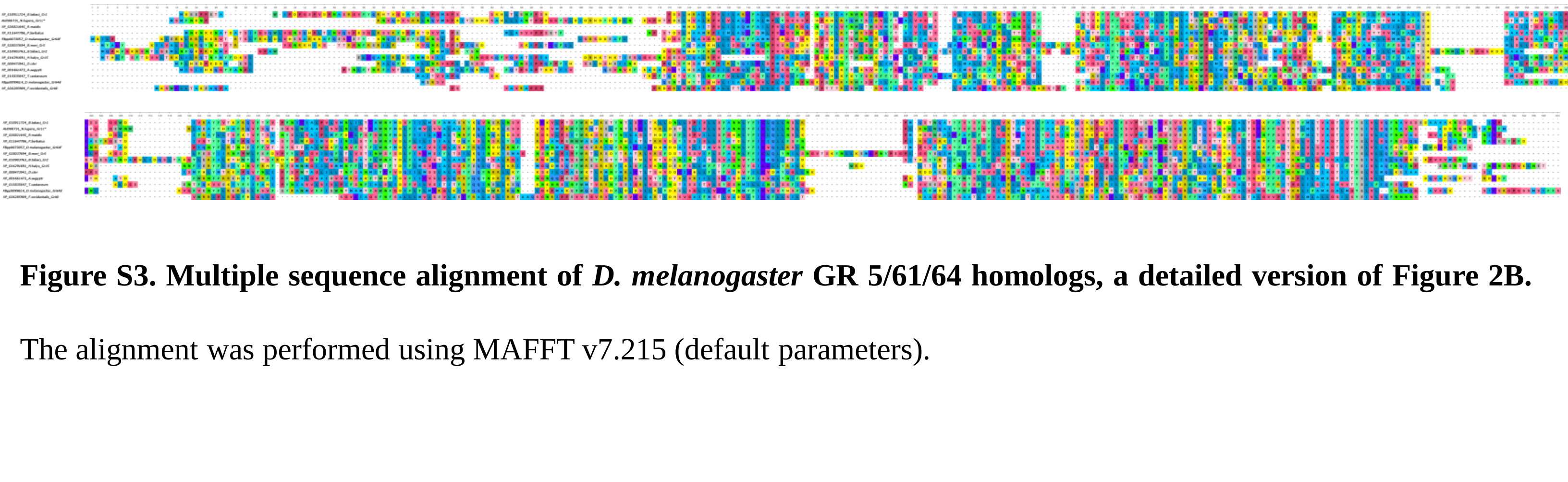

Figure S3. Multiple sequence alignment of *D. melanogaster* GR 5/61/64 homologs, a detailed version of Figure 2B.

The alignment was performed using MAFFT v7.215 (default parameters).

	experiment	P value	rejected	corrected
0	10 vs 10	0.882501	FALSE	0.882501
1	10 vs 50	0.000775	TRUE	0.00142
2	25 vs 25	0.158653	FALSE	0.218148
3	25 vs 75	8.44E-06	TRUE	3.09E-05
4	50 vs 50	0.291	FALSE	0.3201
5	50 vs 300	7.05E-16	TRUE	7.76E-15
6	75 vs 75	0.235464	FALSE	0.28779
7	300 vs 300	0.117419	FALSE	0.184516
8	H2O vs 10	3.50E-05	TRUE	9.64E-05
9	H2O vs 25	1.15E-06	TRUE	6.34E-06
10	H2O vs 50	0.000345	TRUE	0.000758

Table S1 – Statistical details of all conducted choice comparisons, related to Figure 1C.

Indicated name in the tree	Species full name	Order (farther relevant taxonomy group information)
T.castaneum	Tribolium castaneum	Coleoptera (Tenebrionidae)
A.aegypti	Aedes aegypti	Diptera (Culicidae)
D.melanogaster	Drosophila melanogaster	Diptera (Drosophilidae)
L.striatellus	Laodelphax striatellus	Hemiptera (Auchenorrhyncha)
N.lugens	Nilaparvata lugens	Hemiptera (Auchenorrhyncha)
H.halys	Halyomorpha halys	Hemiptera (Heteroptera)
B.tabaci	Bemisia tabaci MEAM1	Hemiptera (Sternorrhyncha:Aleyrodidae)
A.pisum	Acyrthosiphon pisum	Hemiptera (Sternorrhyncha:Aphididae)
A.craccivora	Aphis craccivora	Hemiptera (Sternorrhyncha:Aphididae)
A.glycines	Aphis glycines	Hemiptera (Sternorrhyncha:Aphididae)
A.gossypii	Aphis gossypii	Hemiptera (Sternorrhyncha:Aphididae)
A.lucorum	Apolygus lucorum	Hemiptera (Sternorrhyncha:Aphididae)
C.cedri	Cinara cedri	Hemiptera (Sternorrhyncha:Aphididae)
D.noxia	Diuraphis noxia	Hemiptera (Sternorrhyncha:Aphididae)
M.sacchari	Melanaphis sacchari	Hemiptera (Sternorrhyncha:Aphididae)
M.persicae	Myzus persicae	Hemiptera (Sternorrhyncha:Aphididae)
R.maidis	Rhopalosiphum maidis	Hemiptera (Sternorrhyncha:Aphididae)
S.flava	Sipha flava	Hemiptera (Sternorrhyncha:Aphididae)
D.citri	Diaphorina citri	Hemiptera (Sternorrhyncha:Liviidae)
P.solenopsis	Phenacocus solenopsis	Hemiptera (Sternorrhyncha:Pseudococcidae)
H.laboriosa	Habropoda laboriosa	Hymenoptera (apidae)
C.cinctus	Cephus cinctus	Hymenoptera (Cephidae)
A.mellifera	Apis mellifera	Hymenoptera (Formicidae)
H.saltator	Harpegnathos saltator	Hymenoptera (Formicidae)
O.biroi	Ooceraea biroi	Hymenoptera (Formicidae)
T.curvispinosus	Temnothorax curvispinosus	Hymenoptera (Formicidae)
V.emeryi	Vollenhovia emeryi	Hymenoptera (Formicidae)
P.barbatus	Pogonomyrmex barbatus	Hymenoptera (Orussidae)
P.gracilis	Pseudomyrmex gracilis	Hymenoptera (Orussidae)
N.vitripennis	Nasonia vitripennis	Hymenoptera (Pteromalidae)
N.lecontei	Neodiprion lecontei	Hymenoptera (Symphyta)
A.rosae	Athalia rosae	Hymenoptera (Tenthredinidae)
T.chilonis	Trichogramma chilonis	Hymenoptera (Trichogrammatidae)
B.mori	Bombyx mori	Lepidoptera (Bombycidae)
H.armigera	Helicoverpa armigera	Lepidoptera (Noctuoidea)
P.xylostella	Plutella xylostella	Lepidoptera (Plutellidae)
F.occidentalis	Frankliniella occidentalis	Thysanoptera (Thripidae)

Table S2 - An index of the species in the phylogeny, related to figure 2A. Detailing the full species name and phylogeny of each species appears in Fig 2 and Fig S1

Table S3. List of used carbohydrates. Related to Figure 3.

Sugars	IUPAC name	Haworth projection	CAS No.
Raffinose	(2R,3R,4S,5S,6R)-2-{[(2S,3S,4S,5R)-3,4-Dihydroxy-2,5-bis(hydroxymethyl)oxolan-2-yl]oxy}-6-({[(2S,3R,4S,5R,6R)-3,4,5-trihydroxy-6-(hydroxymethyl)oxan-2-yl]oxy}methyl)oxane-3,4,5-triol	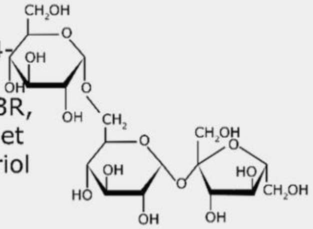	17629-30-0
D-(+)-Sucrose	β-D-Fructofuranosyl α-D-glucopyranoside	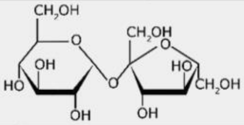	57-50-1
D-(+)-Maltose	(3R,4R,5S,6R)-6-(hydroxymethyl)-5-{[(2R,3R,4S,5S,6R)-3,4,5-trihydroxy-6-(hydroxymethyl)oxan-2-yl]oxy}oxane-2,3,4-triol	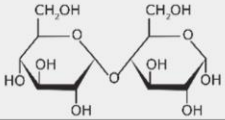	6363-53-7
D-Trehalose	(2R,3S,4S,5R,6R)-2-(Hydroxymethyl)-6-[(2R,3R,4S,5S,6R)-3,4, 5-trihydroxy-6-(hydroxymethyl)oxan-2-yl]oxyoxane-3,4,5-triol	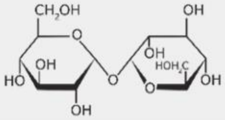	6138-23-4
Lactose monohydrate	(2R,3R,4S,5R,6S)-2-(hydroxymethyl)-6-[(2R,3S,4R,5R,6S)-4,5,6-trihydroxy-2-(hydroxymethyl)oxan-3-yl]oxyoxane-3,4,5-triol;hydrate	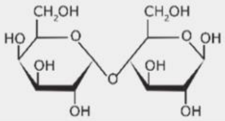	10039-29-6
D-(+)-Glucose	(2R,3S,4R,5R)-2,3,4,5,6-Pentahydroxyhexanal	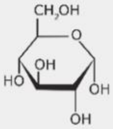	50-99-7
D-(−)- Fructose	(3S,4R,5R)-1,3,4,5,6-Pentahydroxyhexan-2-one	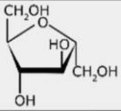	57-48-7
D-(+)-Galactose	(3R,4S,5R,6R)-6-(hydroxymethyl)oxane-2,3,4,5-tetrol	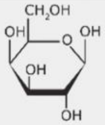	59-23-4
Myo-inositol	(1R,2S,3r,4R,5S,6s)-Cyclohexane-1,2,3,4,5,6-hexol	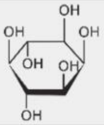	87-89-8
D-Mannose	D-manno-hexopyranose	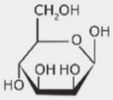	3458-28-4
L-Rhamnose	(2R,3R,4R,5R,6S)-6-Methyloxane-2,3,4,5-tetrol	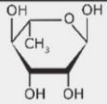	10030-85-0
D-Mannitol	(2R,3R,4R,5R)-Hexane-1,2,3,4,5,6-hexol	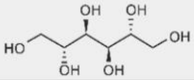	69-65-8
D-Sorbitol	(2S,3R,4R,5R)-Hexane-1,2,3,4,5,6-hexol	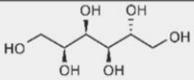	50-70-4
Glycerol	Propane-1,2,3-triol	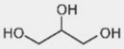	56-81-5

Organism (order)	Receptor	Detected sugars	Methods (paper)
Drosophila melanogaster (Diptera)	DmelGr5a	Tuned to Trehalose rsponse to some extent to melezitose, m-glucoside	Neuron response recordings and Calcium imaging (Chyb et al., 2003; Freeman et al., 2014)
	DmelGr64a	glucose, sucrose, maltose, maltotriose, fructose, glycerol	Neuron response recordings (Freeman et al., 2014)
	DmelGr64b	trehalose, melezitose, m-glucoside, glucose	Neuron response recordings (Freeman et al., 2014)
	DmelGr64c	sucrose, maltose, maltotriose ,fructose	Neuron response recordings (Freeman et al., 2014)
	DmelGr64d	maltose, fructose	Neuron response recordings (Freeman et al., 2014)
	DmelGr64e	trehalose, melezitose	Neuron response recordings (Freeman et al., 2014)
	DmelGr64f	trehalose, melezitose, m-glucoside	Neuron response recordings (Freeman et al., 2014)
	DmelGr61a	m-glucoside, glucose	Neuron response recordings (Freeman et al., 2014)
	DmelGr43a	Narrowly tuned D-fructose receptor	Calcium imaging (Miyamoto et al., 2012)
Spodoptera litura (Lepidoptera)	SlitGR8	Narrowly tuned D-fructose receptor	Xenopus oocyte system (X., L., Liu et al., 2019)
Trichogramma chilonis (Hymenoptera)	TchiGR43a	Narrowly tuned D-fructose receptor	Xenopus oocyte system (J., Liu et al., 2019)
	TchiGR64f1+ TchiGR64f2	Highly sensitive to sucrose, also reactive to glucose, somewhat reactive to additional sugars.	Xenopus oocyte system (J., Liu et al., 2020)
Plutella xylostella (Lepidoptera)	PxylGR43a	Narrowly tuned D-fructose receptor	Xenopus oocyte system (X., L., Liu et al., 2020)
Helicoverpa armigera (Lepidoptera)	HarmGr4	Narrowly tuned D-fructose receptor	Xenopus oocyte system (Jiang et al., 2015)
	HarmGr9	D-fructose, D-galactose, D-maltose	insect cell (Sf9) Calcium imaging system (Xu et al., 2012)
Bombyx mori (Lepidoptera)	BmGr8	responds to myo-inositol and, to a<br>lesser extent, epi-inositol in vitro	Xenopus oocyte system (Zhang et al., 2011)
	BmGr9	Narrowly tuned fructose receptor	Xenopus oocyte system, Calcium imaging (Sato et al., 2011)
	BmGr10	myo-inositol and epi-inositol	Xenopus oocyte system (Kikuta et al., 2016)
Nilaparvata lugens (Hemiptera)	NlGr11	fructose, galactose and arabinose glucose and xylose also elicited response.	insect cell (Sf9) Calcium imaging system (Chen et al., 2019)
Tribolium castaneum	TcGr20	Mannitol, sorbitol	Xenopus oocyte system (Takada et al., 2017)
Apis melifera (Hymenoptera)	AmGr1	sucrose, glucose, trehalose, and maltose	Xenopus oocyte system (Jung et al., 2015)
	AmGr1+AmGr2	higher sensitivity to glucose and lower sensitivity to sucrose, trehalose, and maltose compared with AmGr1 expression alone	

Table S4. Functionally characterized insect GRs, related to Figure 2. Summarizing table of all current functionally characterized insect GRs, including: the insect species, nomenclature, analyzed substrates, and citation.

#	Name	Sequence (5' -> 3')
1	qRT-PCR:Bta04282 (RPL13A) Forward	CATTCCACTACAGAGCTCCA
2	qRT-PCR:Bta04282 (RPL13A) Reverse	TTTCAGGTTTCGGATGGCTT
3	qRT-PCR:Bta10134 (sweet GR) Forward	GTGGACTTCGTGTTTGTGAC
4	qRT-PCR:Bta10134 (sweet GR) Reverse	CTCCGCCTTCTCCCATTG
5	Bta10134 Over intron 7 Forward	CTCCAATAGTTCTCCTATCGTTTGCG
6	Bta10134 Over intron 7 Reverse	GTATGTTGCTTGCCATGTGCTTTG

Table S5. List of primers used in the qRT-PCR and PCR analyses, related to Figure 4B.

Non-essential amino acids (mol%)		Essential amino acids (mol%	
Gly	0.70%	His	0.40%
Ala	9.80%	Ile	1.20%
Arg	1.60%	Leu	1.20%
Asn	10.20%	Lys	1.80%
Asp	9.60%	Met	0.70%
Gln	33.10%	Phe	1.10%
Glu	11.40%	Thr	5.70%
Ser	7.50%	Trp	0.90%
Tyr	0.60%	Val	2.40%
Sum:	84.50%	Sum:	15.50%

Table S6 - The protocol for preparing the amino acids mixture, related to Figure 1 and 4.
